# The log odds of negative lymph nodes/T stage: a new prognostic and predictive tool for resected gastric cancer patients

**DOI:** 10.1007/s00432-021-03654-y

**Published:** 2021-05-18

**Authors:** Jiebin Xie, Yueshan Pang, Xun Li, Xiaoting Wu

**Affiliations:** 1 Department of Gastrointestinal Surgery, West China Hospital, Sichuan University, Chengdu, Sichuan China; 2 Department of Gastrointestinal Surgery, Affiliated Hospital of North Sichuan Medical College, Nanchong, Sichuan China; 3 Department of Geriatrics, The Second Clinical Medical College of North Sichuan Medical College, Nanchong Central Hospital, Nanchong, Sichuan China

**Keywords:** Nomogram, Gastric cancer, Negative lymph nodes, Negative lymph nodes/T stage, Prognosis

## Abstract

**Purpose:**

When only the TNM classification is used to predict survival in gastric cancer (GC) patients, the impact of the degree of lymphadenectomy on the prognosis is neglected. This study aimed to establish a more effective nomogram based on the log odds of negative lymph nodes/T stage ratio (LONT) to predict survival in surgically treated GC patients.

**Methods:**

The data of resected GC patients were extracted from the Surveillance, Epidemiology, and End Results Program (SEER) database. Univariate and multivariate Cox regression analyses were used to identify the significant prognostic factors. The prognostic performance was assessed using a calibration plot, concordance index (C-index), and area under the (time-dependent receiver operating characteristic) curve (AUC) to compare the predicted survival probability based on the nomogram score groups.

**Results:**

The results showed LONT as an independent prognostic factor for cancer-specific survival (CSS) and overall survival (OS), independent of clinicopathological factors. After removing potential redundancy, only LONT, T stage, N stage, location and age were used in the final nomogram model. The model had a higher C-index (0.736 ± 0.012) and AUC (0.798) than the TNM staging system (0.685 ± 0.012 and 0.744). The nomogram score could predict a significant survival difference between any two adjacent groups in terms of CSS and OS.

**Conclusion:**

High LONT is associated with improved survival of gastric cancer patients, independent of other clinicopathological factors. The prognostic nomogram model based on LONT could effectively predict CSS and OS for resectable GC patients.

**Supplementary Information:**

The online version contains supplementary material available at 10.1007/s00432-021-03654-y.

## Introduction

Gastric cancer (GC) is the third most common cause of cancer death and the fifth most common cancer globally, with over 1 million estimated new cases annually (Smyth et al. 2020). GC is therefore regarded as a major global health problem, especially in East Asian countries (GBD 2017; Stomach Cancer Collaborators 2020). The accuracy of survival prediction for GC patients is crucial for postoperative treatment and follow-up plans. To date, tumor-node-metastasis (TNM) staging based on the depth of tumor invasion and the number of regional positive lymph nodes is the most widely accepted system for risk stratification in GC (Fujitani et al. 2018). However, the clinical outcomes among GC patients with the same TNM stage might be completely different. The conflicting results might be because the system is based only on the extent of disease and disregards the influence of the degree of lymph node dissection (LND) on survival.

Presently, the evaluation of the degree of LND mainly depends on the extent of lymph nodes removed at the time of gastrectomy (Japanese Gastric Cancer Association 2020). Although D2 LND describes the extent of lymphadenectomy with the goal of examining more than 16 lymph nodes (Degiuli et al. 2004; Schwarz and Smith 2007; Son et al. 2012), the difference in the technical aspects of performing D2 LND may lead to different survival (Enzinger et al. 2007; Liang et al. 2015). Moreover, this model has inherent limitations; namely, the evaluation by the surgeon is considered, not the pathologist, and it lacks objective quantitative indicators. In recent years, observational studies have indicated that the count of examined lymph nodes (ELNs) (Smith et al. 2005; Son et al. 2012) and negative LNs (NLNs) (Kattan et al. 2003; Martinez-Ramos et al. 2014; Wang et al. 2017) have independent prognostic value in GC, which can reflect the degree of LND. However, they also have limitations due to the lack of information on individualized tumor characteristics.

Indeed, T stage is a robust risk factor in GC, which is based on the depth of tumor invasion and can represent the major tumor characteristics. Increasing studies have shown that T stage is not only related to prognosis but also closely related to tumor biological characteristics (Mao et al. 2019; Sun et al. 2020; Wang et al. 2019). Both NLNs and T stage are important independent prognostic factors in GC, which represent the degree of LND and the severity of disease, respectively. However, whether the combination of NLNs and T stage can serve as a novel prognostic factor that reflects the degree of individualized LND in GC patients remains unclear. Thus, in our study, we first defined the log odds of negative lymph nodes/T stage (LONT) as log^(NLNs+1)/T stage^, which represents the NLNs adjusted by the T stage, to better reflect the degree of LND.

Here, we used a population-based cohort from the Surveillance, Epidemiology, and End Results (SEER) database and aimed to investigate the correlation between LONT and prognosis. Based on LONT, we also constructed a novel prognostic nomogram model to predict survival in surgically treated GC patients.

## Materials and methods

### Patients

The GC patients were screened from the SEER database using SEER*Stat 8.3.8 software. We used the primary site codes C16.0-C16.9 for gastric and the International Classification of Diseases for Oncology, Third Edition (ICD-O-3) histologic codes 8140/3 (adenocarcinoma, NOS), 8144/3 (adenocarcinoma, intestinal type), 8211/3 (tubular adenocarcinoma), 8255/3 (adenocarcinoma with mixed subtypes), 8260/3 (papillary adenocarcinoma), 8263/3 (adenocarcinoma, tubulovillous adenoma), 8480/3 (mucinous adenocarcinoma), 8490/3 (signet-ring cell carcinoma) for adenocarcinoma(Zhu et al. 2020). Patients with only one primary malignancy who were pathologically confirmed were included. Finally, we identified a total of 55,536 cases between 2004 and 2017.

Information on surgery type, exact tumor size, location, regional nodes examined, regional nodes positive, tissue type, tumor differentiation, CS extension, survival status, and demographic characteristics was collected. The TNM status of each patient was re-evaluated according to the 8th edition of the American Joint Committee on Cancer (AJCC) Cancer Staging Manual based on CS extension and regional nodes positive in the SEER database. Patients with stage I to III GC who did not undergo radical excision or lacked a detailed description of the surgery, were less than 18 years old, or contained any missing data for selected variables were excluded.

The included patients were randomly divided into the training cohort and the validation cohort (7:3) for cross-validation. The cases excluded due to unknown tumor size, race, differentiation, or location were used to assess the robustness of the nomogram. The training cohort was used to develop the prognostic nomogram model, and the validation cohort and the missing data cohort were used to validate the model.

### Statistical analysis and nomogram development

In our study, T1, T2, T3, T4a, and T4b were assigned 1, 2, 3, 4, and 5, respectively. LONT was analyzed as a continuous variable in the study, which was defined as log^(NLNs+1)/T stage^, where NLNs is the ELNs minus the count of positive LNs. One is added for NLNs to avoid the occurrence of zero (Wen et al. 2017). Continuous and categorical variables are expressed as medians and totals (percentages), respectively. cancer-specific survival (CSS) was the primary endpoint, and cases in which the cause of death was unclear or death was due to other causes were treated as censored observations. Overall survival (OS).

Univariate and multivariate logistic regression models were performed to identify variables that were independently associated with CSS and OS. Stratified analysis of the LONT effect on CSS and OS based on different clinicopathological factors was also performed by Cox proportional hazards regression. A time-dependent receiver operating characteristic curve (ROC) and concordance index (C-index) were utilized to evaluate the discriminative ability of LONT and other factors and used to further exclude unnecessary variables. Then, according to the ROC and C-index results, we chose the simplest combination of variables and built the final nomogram.

### Model validation

To validate our model, we used the following four criteria to assess the prediction performance in both validation cohorts (Wang et al. 2018). First, calibration curves were plotted to judge the consistency between predicted survival probability and actual survival proportion at 3, 5, and 10 years for CSS and OS, respectively. Second, the cases were grouped according to their total nomogram score, and Kaplan–Meier survival analysis was used to plot the survival curves and to compare the survival rate among the different groups by log-rank test. Third, the AUC of the time-dependent ROC curve was calculated at 5 years and compared with the model with the 8th TNM stage. Fourth, the C-index was used to evaluate the prediction accuracy of the model, with a larger C-index value indicating better accuracy.

Nomogram development and validation were performed using RStudio software (version 3.6.3). Other analyses were performed using SPSS (version 22.0). A two-tailed *P* value less than 0.05 was considered statistically significant.

## Results

### Baseline characteristics

**Fig. 1:**
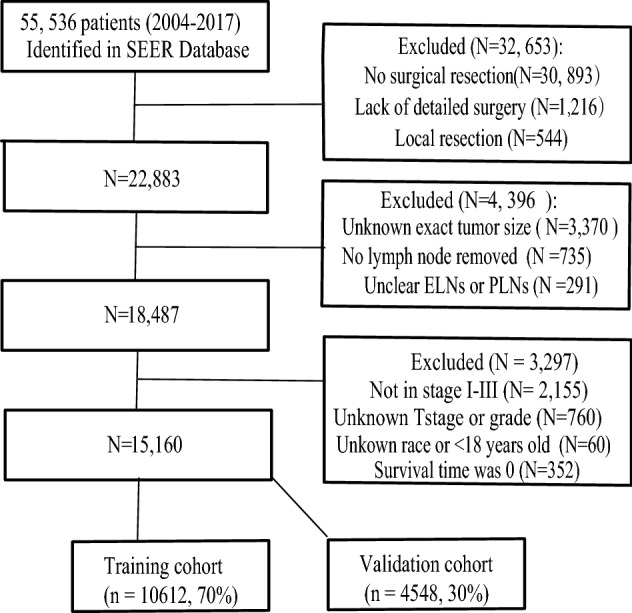
Flowchart of patient selection

The data of 55,536 patients pathologically diagnosed with primary GC from 2004 to 2017 were obtained from the SEER database. Among these patients, 32,653 did not undergo surgical resection, or details about their surgery were lacking. Information regarding the exact tumor size was not available for 3370 patients. In addition, 1026 patients were excluded because no LNs were removed or information regarding ELNs or PLNs was incomplete. Of the remaining 18,487 patients, 2342 did not have stage I/III disease (2155) or had an unknown T stage (187), 573 had an unknown grade, 57 were of an unknown race, and 3 were less than 18 years old. Finally, 352 lacked information regarding survival time. Thus, a total of 15,160 patients were ultimately included and randomly divided into two cohorts (Fig. 1): the training cohort (*n* = 10,612, 70%) and the validation cohort (*n* = 4548, 30%). In addition, 2799 patients with missing information on tumor size, race, differentiation, or location data constituted the missing data cohort (Table S1). The latest follow-up date was in November 2019. The median follow-up time was 30 months (range 1–167 months) in the training cohort and 29 months (range 1–167 months) in the validation cohort. The clinicopathological characteristics between the two cohorts were similar, but the proportion of patients with stage III disease in the validation cohort was significantly higher than that in the training cohort (*P* = 0.013). Detailed information about the clinicopathological features is shown in Table 1.

**Table 1:** Baseline clinicopathological characteristics of patients in the training and validation cohort

Feature	Training cohort (*N* = 10,612)	Validation cohort (*N* = 4548)	*P*
	No. of patients (%)	No. of patients (%)	
Median LONT (IQR)	0.7 (0.4–1.0)	0.7 (0.4–1.0)	0.414
Median size (IQR) mm	40 (25–60)	40 (25–60)	0.167
Median PLN count (IQR)	1 (0–5)	1 (0–5)	0.938
Median ELN count (IQR)	16 (9–24)	16 (10–24)	0.341
Median NLN count (IQR)	12 (6–20)	12 (6–20)	0.291
Age (years)			0.915
< 60	3061 (28.8)	1264 (27.8)	
≥ 60	7551 (71.2)	3284 (72.2)	
Sex			0.450
Male	6792 (64)	2881 (63.3)	
Female	3820 (36.0)	1667 (36.7)	
TNM stage			0.013
I	31.26 (29.5)	1356 (29.8)	
II	3443 (32.4)	1370 (30.1)	
III	4043 (38.1)	1822 (40.1)	
Differentiation			0.779
Poor/anaplastic (G3 + 4)	6831 (64.4)	2901 (63.8)	
Moderate (G2)	3209 (30.2)	1395 (30.7)	
High (G1)	572 (5.4)	252 (5.5)	
T stage			
T1	2474 (23.3)	1034 (22.7)	0.216
T2	1496 (14.1)	670 (14.7)	
T3	4129 (38.9)	1798 (39.5)	
T4a	1950 (18.4)	841 (18.5)	
T4b	563 (5.3)	205 (4.5)	
N stage			0.425
N0	4766 (44.9)	1995 (43.9)	
N1	1975 (18.6)	832 (18.3)	
N2	1764 (16.6)	808 (17.8)	
N3a	1499 (14.1)	657 (14.4)	
N3b	608 (5.7)	256 (5.6)	
Location			0.805
Cardiac/fundus	3165 (29.8)	453 (10.0)	
Body	989 (9.3)		
Lesser/greater curvature	1742 (16.4)	742 (16.3)	
Antrum/pylorus	3360 (31.7)	1422 (31.3)	
Other	1356 (12.8)	573 (12.6)	
Histology type			0.997
Adenocarcinoma	8143 (76.7)	3490 (76.7)	
MAC and SRCC	2469 (23.3)	1058 (23.3)	
Race			0.392
White	6852 (64.6)	2988 (65.7)	
Black	1387 (13.1)	582 (12.8)	
Others	2373 (22.4)	978 (21.5)	

*ELNs* examined lymph nodes, *IQR* interquartile range, *LONT* Log odds of negative lymph nodes/T stage, *MAC* mucinous adenocarcinoma, *NLNs* negative lymph nodes, SRCC signet-ring cell carcinoma

### The prognostic impact of LONT on CSS and OS

In univariate analysis, all the included variables were significantly correlated with CSS (Table 2) and OS (Table S2) in the training group. Except for sex on CSS (Table 2) and histology type on OS (Table S2), the other variables had similar results in the validation cohort. To avoid losing prognostic information, we used T stage and N stage instead of TNM stage for the multivariate analysis. The results showed that LONT, age, race, T stage, N stage, and location but sex and histology type were independent prognostic factors for CSS (Table 3) and OS (Table S3) in both cohorts, while tumor size for CSS and grade for OS were not significantly associated with the survival outcome in the validation subset. To confirm the independent prognostic effect of LONT, the prognostic impact of LONT on CSS and OS was further examined by stratified analysis with a multivariate Cox proportional hazards model. According to the results, all the subsets were significantly associated with CSS (Table 4) and OS (Table S4) in both cohorts. All the results showed that lower LONT values indicated a worse prognosis.

**Table 2:** Univariate analysis of included variables on cancer-specific survival

Variable	Training cohort (*n* = 10,612)			Validation cohort (*n* = 4548)		
	HR	95% CI	*P*	HR	95% CI	*P*
Tumor size^a^	1.003	1.003–1.003	< 0.001	1.003	1.002–1.003	< 0.001
LONT^a^	0.255	0.24–0.272	< 0.001	0.276	0.252–0.302	< 0.001
NLNs^a^	0.965	0.961–0.968	< 0.001	0.966	0.961–0.971	< 0.001
Age(ref = > 60)	0.873	0.817–0.932	< 0.001	0.855	0.772–0.946	0.002
Sex (ref = male)	0.936	0.880–0.995	0.035	0.941	0.857–1.034	0.205
TNM (ref = stage III)			< 0.001			< 0.001
I	0.149	0.135–0.164	< 0.001	0.160	0.139–0.185	< 0.001
II	0.411	0.384–0.439	< 0.001	0.475	0.429–0.526	< 0.001
T stage ( ref = T4b)			< 0.001			< 0.001
T1	0.129	0.112–0.148	< 0.001	0.136	0.108–0.171	< 0.001
T2	0.255	0.222–0.293	< 0.001	0.268	0.215–0.334	< 0.001
T3	0.533	0.478–0.594	< 0.001	0.567	0.473–0.681	< 0.001
T4a	0.830	0.741–0.931	< 0.001	0.858	0.709–1.039	0.116
N stage ( ref= N3b)			< 0.001			< 0.001
N0	0.145	0.130–0.162	< 0.001	0.157	0.133–0.187	< 0.001
N1	0.330	0.295–0.370	< 0.001	0.375	0.315–0.446	< 0.001
N2	0.474	0.424–0.530	< 0.001	0.480	0.404–0.570	< 0.001
N3a	0.666	0.596–0.744	< 0.001	0.772	0.651–0.915	0.003
Differentiation (ref = G3 + G4)						
G1	0.368	0.308–0.441	< 0.001	0.455	0.354–0.586	< 0.001
G2	0.603	0.562–0.646	< 0.001	0.671	0.606–0.744	< 0.001
Histology(ref = adenocarcinoma)	1.281	1.198–1.369	< 0.001	1.196	1.079–1.325	0.001
Location (ref = cardiac)						
Middle^b^	0.742	0.685–0.804	< 0.001	0.822	0.731–0.924	0.001
Antrum/pylorus	0.833	0.772–0.898	< 0.001	0.757	0.673–0.851	< 0.001
Other	1.092	0.994–1.201	0.067	0.968	0.835–1.121	0.661
Race (ref = others)			< 0.001			< 0.001
White	1.359	1.258–1.467	< 0.001	1.364	1.211–1.536	< 0.001
Black	1.348	1.213–1.498	< 0.001	1.398	1.190–1.643	< 0.001

*CI* Confidence interval, *HR* Hazard ratio, *LONT* Log odds of negative lymph nodes/T stage, *NLNs* negative lymph nodes, *ref* Reference

^a^These variables were treated as continuous data

^b^The location included the body, fundus, greater curvature and lesser curvature of the stomach.

**Table 3:** Multivariate analysis of prognostic factors for cancer-specific survival

Variable	Training cohort (*n* = 10,612)			Validation cohort (*n* = 4548)		
	HR	95% CI	*P*	HR	95% CI	*P*
Age(ref = ≧ 60 years old)	0.699	0.654–0.748	< 0.001	0.734	0.662–0.813	< 0.001
Tumor size^a^	1.001	1.001–1.002	< 0.001	1.001	1.00–1.002	0.060
LONT^a^	0.469	0.435–0.505	< 0.001	0.489	0.437–0.547	< 0.001
Sex (ref = male)	0.988	0.927–1.053	0.599	0.989	0.897–1.090	0.820
T stage (ref= T4b)						
T1	0.405	0.345–0.476	< 0.001	0.416	0.320–0.540	< 0.001
T2	0.510	0.440–0.591	< 0.001	0.541	0.427–0.687	< 0.001
T3	0.722	0.645–0.809	< 0.001	0.744	0.614–0.902	0.003
T4a	0.855	0.761–0.959	< 0.001	0.891	0.734–1.083	0.247
N stage (ref= N3b)						
N0	0.294	0.260–0.332	< 0.001	0.295	0.244–0.356	< 0.001
N1	0.492	0.436–0.555	< 0.001	0.496	0.414–0.595	< 0.001
N2	0.616	0.548–0.691	< 0.001	0.571	0.478–0.681	< 0.001
N3a	0.766	0.684–0.857	< 0.001	0.844	0.710–1.004	0.056
Differentiation (ref = G3 + G4)						0.035
G1	0.744	0.618–0.894	< 0.001	0.856	0.658–1.112	0.245
G2	0.801	0.744–0.863	< 0.001	0.871	0.780–0.971	0.013
Histology (ref = adenocarcinoma)	1.035	0.962–1.113	0.356	1.007	0.903–1.124	0.900
Location (ref = cardiac)						
Middle^b^	0.645	0.593–0.702	< 0.001	0.727	0.641–0.824	< 0.001
Antrum/pylorus	0.697	0.642–0.756		0.674	0.595–0.763	< 0.001
Other	0.737	0.667–0.814	0.043	0.719	0.617–0.838	< 0.001
Race (ref = others)						
White	1.257	1.161–1.361	< 0.001	1.182	1.046–1.336	0.007
Black	1.320	1.187–1.468	< 0.001	1.279	1.086–1.507	0.003

*CI* Confidence interval, *HR* Hazard ratio, *LONT* Log odds of negative lymph nodes/T stage, *ref*: Reference

^a^These variables were treated as continuous data

^b^The location included the body, fundus, greater curvature and lesser curvature of the stomach

**Table 4:** Multivariate analyses for evaluating the LONT effect on cancer-specific survival based on different clinicopathological factors^1^

Variable	Training cohort (*n* = 10,612)			Validation cohort (*n* = 4548)		
	HR	95% CI	*P*	HR	95% CI	*P*
All	0.455	0.427–0.496	< 0.001	0.446	0.401–0.496	< 0.001
N stage						
N0	0.372	0.313–0.442	< 0.001	0.447	0.342–0.584	< 0.001
N1	0.389	0.330–0.459	< 0.001	0.459	0.361–0.583	< 0.001
N2	0.409	0.351–0.476	< 0.001	0.464	0.370–0.581	< 0.001
N3a	0.482	0.420–0.554	< 0.001	0.435	0.351–0.538	< 0.001
N3b	0.552	0.454–0.669	< 0.001	0.347	0.251–0.482	< 0.001
T stage						
T1	0.385	0.287–0.517	< 0.001	0.431	0.272–0.682	< 0.001
T2	0.426	0.332–0.564	< 0.001	0.395	0.267–0.586	< 0.001
T3	0.432	0.386–0.484	< 0.001	0.468	0.395–0.555	< 0.001
T4a	0.493	0.432–0.563	< 0.001	0.442	0.362–0.540	< 0.001
T4b	0.328	0.260–0.415	< 0.084	0.488	0.330–0.722	< 0.001
TNM stage						
I	0.413	0.323–0.528	< 0.001	0.461	0.319–0.666	< 0.001
II	0.409	0.352–0.475	< 0.001	0.443	0.354–0.555	< 0.001
III	0.442	0.405–0.482	< 0.001	0.453	0.398–0.515	< 0.001
Sex						
Male	0.467	0.427–0.511	< 0.001	0.434	0.380–0.495	< 0.001
Female	0.377	0.336–0.424	< 0.001	0.458	0.381–0.551	< 0.001
Age years						
≤ 60	0.398	0.349–0.454	< 0.001	0.406	0.330–0.499	< 0.001
> 60	0.446	0.409–0.485	< 0.001	0.425	0.399–0.512	< 0.001
Differentiation						
G1	0.462	0.282–0.756	0.002	0.327	0.167–0.641	0.001
G2	0.385	0.329–0.451	< 0.001	0.521	0.418–0.650	< 0.001
G3 + G4	0.443	0.408–0.480	< 0.001	0.432	0.381–0.489	< 0.001
Location						
Cardiac	0.528	0.460–0.605	< 0.001	0.478	0.390–0.585	< 0.001
Non-Cardiac	0.391	0.355–0.430	< 0.001	0.422	0.366–0.486	< 0.001
Race						
White	0.439	0.402–0479	< 0.001	0.451	0.396–0.515	< 0.001
Black	0.408	0.337–0.493	< 0.001	0.495	0.373–0.658	< 0.001
Others	0.440	0.375–0.516	< 0.001	0.387	0.302–0.497	< 0.001
Histology						
Adenocarcinoma	0.424	0.389–0.463	< 0.001	0.445	0.392–0.506	< 0.001
MAC and SRCC	0.453	0.399–0.514	< 0.001	0.459	0.376–0.560	< 0.001
ELNs						
10	0.398	0.332–0.477	< 0.001	0.518	0.395–0.681	< 0.001
11–20	0.333	0.281–0.395	< 0.001	0.265	0.202–0.348	< 0.001
> 20	0.322	0.277–0.375	< 0.001	0.303	0.237–0.387	< 0.001

*CI* Confidence interval, *HR* Hazard ratio, *LONT* Log odds of negative lymph nodes/T stage, *MAC* mucinous adenocarcinoma, *SRCC* signet-ring cell carcinoma

^1^LONT was treated as continuous data, and adjusted by age, race, sex, location, histology type, differention and TNM stage

### Nomogram development in the training cohort

**Fig. 2:**
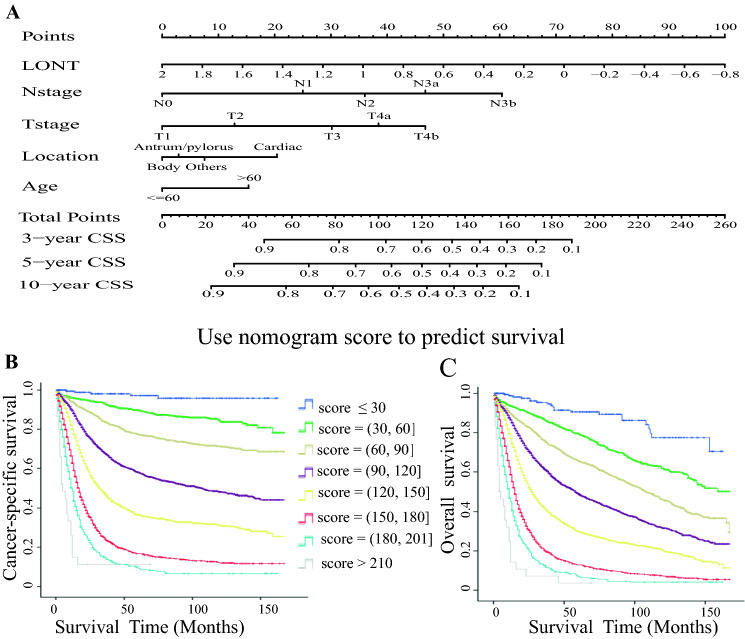
The nomogram and Kaplan–Meier survival plot based on the risk score. **a** The nomogram was applied by adding up the points identified on the points scale for each variable. The sum of these points projected on the bottom scales estimates the probability of 3-, 5- and 10-year CSS. **b** Kaplan–Meier survival analyses for cancer-specific survival based on nomogram scores. **c** Kaplan–Meier survival analyses for overall survival based on nomogram scores

To obtain a simple nomogram for clinical application, a time-dependent ROC and C-index were used to further remove potential redundancy. After removing the factors of size, race, and grade, the C-index and AUC decreased slightly; however, when we removed location and age, especially T stage and N stage, the C-index and AUC all decreased strikingly for CSS (Table 5) and OS (Table S5). Thus, only LONT, N stage, T stage, location and age were used in the final nomogram model. Figure 2 depicts the risk score of each item in the final nomogram. LONT occupied the largest proportion of risk scores, followed by N stage and T stage. Kaplan–Meier survival analysis confirmed that the nomogram risk score had excellent survival prediction ability for CSS (Fig. 2b) and OS (Fig. 2c). The C-index 0.741 (95% CI 0.733–0.749) and AUC (0.810) for the established nomogram were higher than those for the 8th TNM classification (0.691; 95% CI 0.683–0.699; AUC 0.755).

**Table 5:** The discriminatory ability of prognostic factors in predicting cancer-specific survival in the training cohort

Variable	Training cohort (*n* = 10,612)	
	C-index	*AUC*
Age	0.522	0.530
Tumor size	0.627	0.663
LONT	0.689	0.733
TNM	0.691	0.755
T stage	0.673	0.728
N stage	0.681	0.735
Grade	0.570	0.588
Location	0.538	0.556
Race	0.525	0.538
LONT + T stage + N stage + Location + Age + Grade + size + race	0.746	0.812
LONT + T stage + N stage + Location + Age + Grade + size	0.745	0.811
LONT + T stage + N stage + Location + Age + Grade	0.744	0.810
LONT + T stage + N stage + Location + Age	0.741	0.808
LONT + T stage + N stage + Location	0.737	0.806
LONT + T stage + N stage	0.734	0.798
LONT + N stage	0.728	0.788
LONT + T stage	0.711	0.767

*AUC* the area under the time-dependent receiver operating characteristic curve, *C*-*index* concordance index, *LONT* Log odds of negative lymph nodes/T stage

LONT was treated as continuous data

### Nomogram in the validation cohort and the missing data

**Fig. 3:**
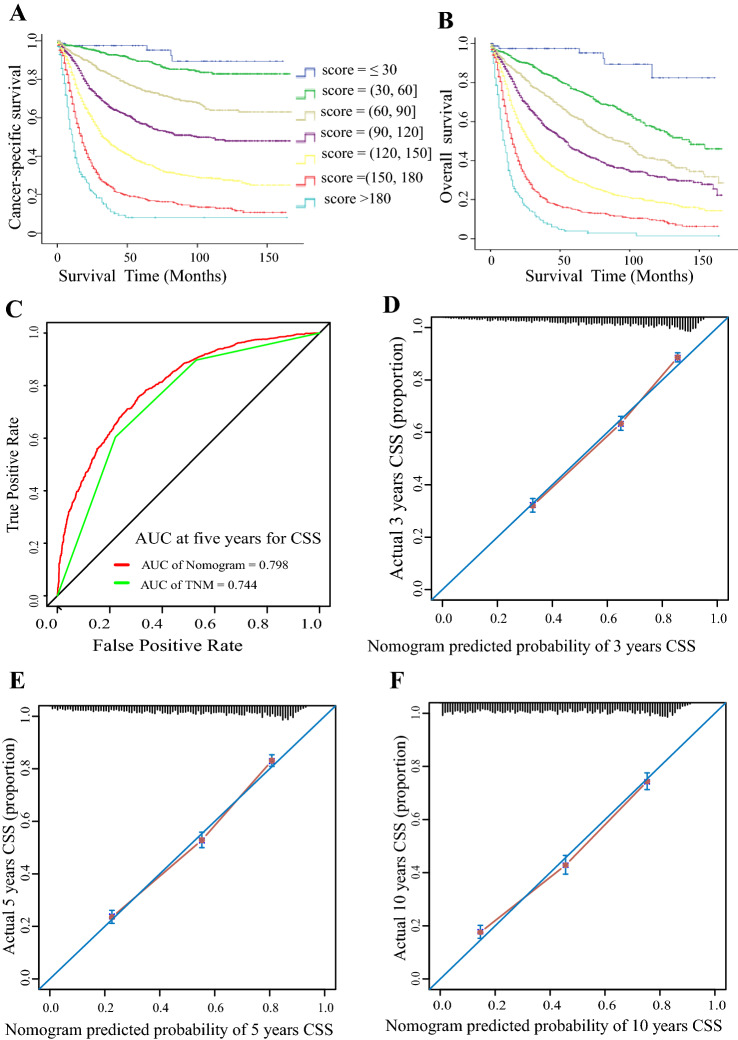
Performance of the prognostic nomogram model in the validation cohort. **a** Kaplan–Meier survival analyses for cancer-specific survival based on nomogram scores, which were calculated according to the nomogram in Fig. 2a. **b** Kaplan–Meier survival analyses for overall survival based on nomogram scores. **c** The area under the time-dependent ROC curve was calculated for the 8th TNM staging system and the nomogram at five years for cancer-specific survival. Red: the nomogram established in the present study; green: the 8th TNM staging system. The calibration curves for predicting patient CSS at 3 years (**d**), 5 years (**e**) and 10 years (**f**). The nomogram model predicting CSS is plotted on the x-axis, and the actual survival proportion is shown on the y-axis

Figures 3a (CSS) and 3B (OS) show that the nomogram risk score can accurately predict the survival difference between any two adjacent groups. The integrated AUC (Fig. 3c) and C-index (0.736; 95% CI 0.724–0.748) for the nomogram were higher than those for the 8th TNM classification (0.685, 95% CI 0.673–0.697). The calibration curve showed that predictions of 3-year (Fig. 3d), 5-year (Fig. 3e), and 10-year (Fig. 3f) CSS were highly consistent with the actual survival proportion. Similar results were observed for OS (Figure S1).

To verify the reliability of the nomogram, the missing data cohort was also used for further verification. The results were highly consistent with the validation group, and the survival difference between any two neighboring risk groups was still significant in both CSS (Figure S2 A) and OS (Figure S2 B) analyses. The values of the C-index (0.751, 95% CI 0.735–0.767) and AUC were even higher than those in the validation cohort (Figure S2C). Similar calibration results also showed that the predicted survival probability at 3, 5, and 10 years for CSS (Figure S2 D, E, F) was highly consistent with the actual survival proportion.

## Discussion

In the present study, we used LONT to quantify the relative degree of LND; moreover, a prognostic nomogram model based on LONT was established and validated using the SEER database. Our results proved that a high LONT was associated with improved survival of GC patients and independent of clinicopathological factors. The nomogram based on LONT, N stage, T stage, location and age not only included information on the tumor characteristics itself but also included information on the degree of LND and exhibited better prognostic performance than TNM stage, which can assist clinicians in developing individualized treatment strategies for GC patients after gastrectomy. To our knowledge, this is the first study quantifying the relative degree of LND and developing a novel nomogram based on LONT and clinicopathological factors to predict the survival of GC patients after gastrectomy.

An accurate prediction of the prognosis of patients with GC plays a very important role in postoperative treatment and follow-up planning. For resectable GC, the prognosis is related not only to the TNM stage (In et al. 2017) and biological characteristics (Mao et al. 2019; Sun et al. 2020; Wang et al. 2019) but also to the degree of LND (Degiuli et al. 2004; Enzinger et al. 2007; Schwarz and Smith 2007) and postoperative comprehensive treatment (Smyth et al. 2020). However, so far, except for the extent of lymphadenectomy at the time of gastrectomy, only ELNs and NLNs can reflect the degree of LND to some extent. However, patients with different disease states have their own individualized optimal ELNs and NLNs; therefore, just using ELNs or NLNs cannot compare the degree of LND among patients with different TNM stages. Overall, we still lack indicators on how to objectively evaluate the degree of LND.

Previous studies have shown that both ELNs (Smith et al. 2005; Son et al. 2012) and NLNs (Kattan et al. 2003; Martinez-Ramos et al. 2014; Wang et al. 2017) are independent prognostic factors for GC. Unfortunately, due to the lack of important information, such as tumor biological characteristics, their clinical value needs further study. In our study, LONT was defined for the first time as the log of the ratio between the NLN counts plus one (Wen et al. 2017) and the T stage; the NLNs represent the total level of LND, and the T stage represents the severity of the disease. The NLNs adjusted by T stage can be understood as the relative number of negative lymph nodes removed for each patient. A higher value indicates that more NLNs were obtained, and conversely, a lower LONT value means fewer NLNs were obtained. Therefore, it can be used to compare the relative level of LND among different patients. Even in patients with different TNM stages, different ELNs or NLNs, the same LONT value represents the same risk level.

In our study, the univariate analyses showed that the HR of NLNs was 0.965 (95% CI 0.961–0.968), and remarkably, that of the NLNs adjusted by T stage was 0.255 (95% CI 0.24–0.272). This confirmed that adjusting for the effect of the T stage may significantly improve the prognostic value of NLNs. A similar result was validated by multivariate Cox analysis and in the validation cohort. The results of subgroup analysis also further confirmed the LONT effect on the CSS and OS rates for different clinicopathological factors. All the results indicated that LONT was an independent prognostic factor for surgically treated GC patients.

To obtain the simplest and most effective nomogram for clinical application, unlike the previously published nomogram including all prognostic factors (Dikken et al. 2013; Kim et al. 2015; Wen et al. 2017). In the present study, ROC and C-index were used to further remove potential redundancy. The results showed that after removing the factors of size, race, and grade, the C-index and AUC decreased only slightly; however, when we removed location and age, especially T stage and N stage, the C-index and AUC all decreased strikingly for CSS (Table 5) and OS (Table S5). Therefore, our nomogram model only included age, location, LONT, N stage, and T stage. The C-index of the nomogram model was 0.741 (95% CI 0.733–0.749) for CSS. Kaplan–Meier survival analysis confirmed the excellent discriminant ability of the model between any neighboring two risk groups for CSS and OS in the validation and missing data cohorts. The value of the C-index and AUC was higher than that of the 8th TNM stage, also indicating the strong predictive ability of our nomogram model (Table 5). Thus, this comprehensive and personalized risk score prediction method could be applied as stratification criteria in guiding postoperative treatment and follow-up planning.

Notably, LONT had the largest proportion of risk scores in the model, with clear risk discriminatory ability for the same T stage, N stage, TNM stage or other clinicopathological factors (Table 3), and showed a higher C-index (0.689 ± 0.008) and AUC (0.733) than T stage (Table 5). The vital contribution of LONT to the nomogram also confirmed the influence of the degree of LND on the prognosis and highlighted the importance of using LONT for prognosis prediction of GC. Moreover, this marker can be simply calculated from the postoperative pathological report at no extra cost. With respect to the prevalence of the model in clinical applications, additional improvements in the accuracy of estimating survival outcomes will benefit more patients.

Using the SEER data empowers us to draw sound conclusions consistent with general clinical practice based on a large sample number of GC patients. However, we must admit that the current study has some inherent limitations. First, we lacked some routinely available clinical parameters, such as lymphovascular invasion, margin status, nerve invasion, CEA, and CA19-9. This information may affect the predictive value of the factors identified in our model. Second, the treatment was not considered because the SEER database did not provide detailed preoperative and postoperative treatment information for these patients. We assume that all patients received the same treatment. Third, the T stage as an indication of tumor characteristics is not accurate because the histological type, differentiation degree and genotyping of GC are also important biological characteristics but were not included in our adjustment. Furthermore, although we used the validation cohort and missing data cohort to verify our model, our results were not validated in our database, and due to the retrospective nature of the SEER and above the limitations, prospective data are needed to confirm these findings.

## Conclusions

In conclusion, LONT is a new prognostic indicator that can reflect the relative degree of LND among different patients. It could effectively predict CSS and OS for resectable GC patients, independent of clinicopathological features. The established nomogram based on LONT showed better discriminatory ability than the 8th TNM staging system, which is a simple, accurate and easy-to-use scoring method for clinicians to develop individualized treatment strategies.

## Supplementary Information

Below is the link to the electronic supplementary material.

Supplementary Figure S1: Performance of the prognostic nomogram model in the validation cohort for OS (PDF 444 KB)

Supplementary Figure S2: Performance of the prognostic nomogram model in the missing data cohort for CSS (PDF 430 KB)

Supplementary Table S1: Baseline clinicopathological features of patients in the excluded cohort and included cohort; Table S2: Univariate analysis of included variables on overall survival; Table S3: Multivariate analysis of prognostic factors for overall survival; Table S4: Multivariate analyses for evaluating LONT effect on overall survival based on different clinicopathological factors; Table S5: Discriminatory ability of prognostic factors in predicting overall survival in the validation cohort. (DOCX 34 KB)
