# Supplementary figures and images for: The log odds of negative lymph nodes/T stage: a new prognostic and predictive tool for resected gastric cancer patients
Source: J Cancer Res Clin Oncol. 2021 May 18;147(8):2259–69. doi: 10.1007/s00432-021-03654-y (PMC8236481; doi:10.1007/s00432-021-03654-y)

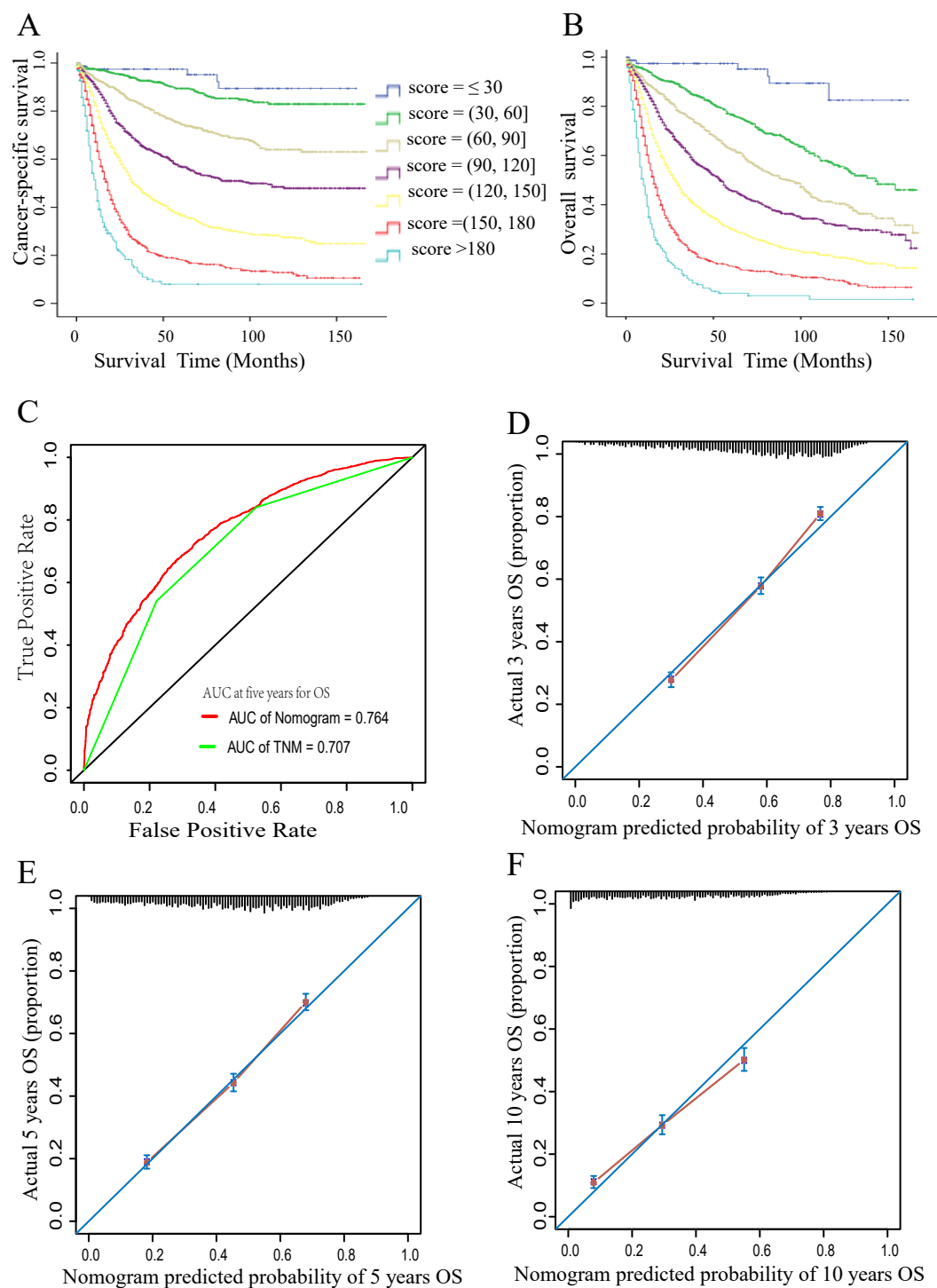

Supplement: Supplementary file 1 — Supplementary Figure S1: Performance of the prognostic nomogram model in the validation cohort for OS (PDF 444 KB) [file 432_2021_3654_MOESM1_ESM.pdf]

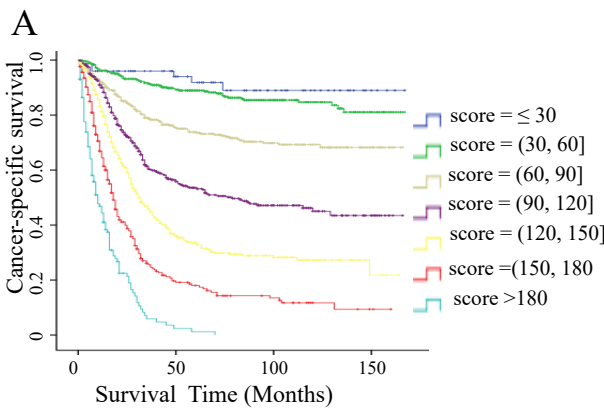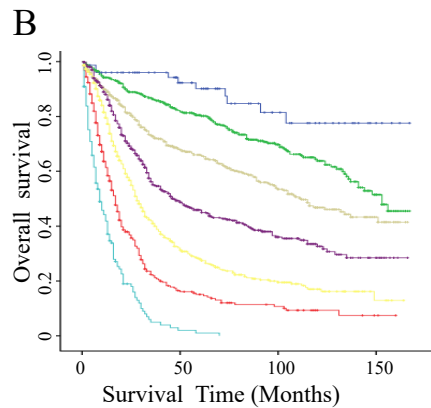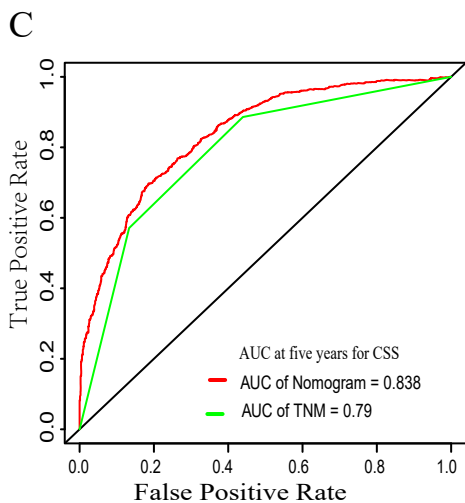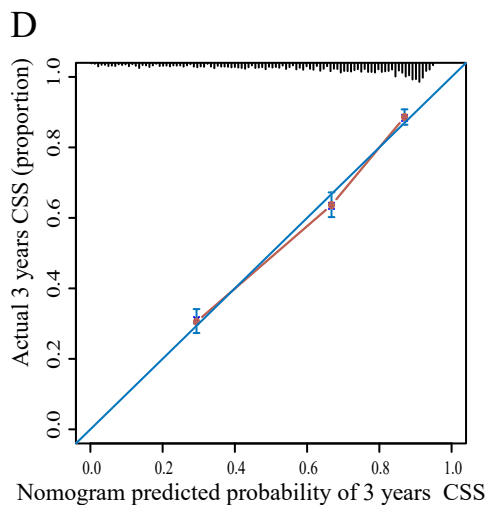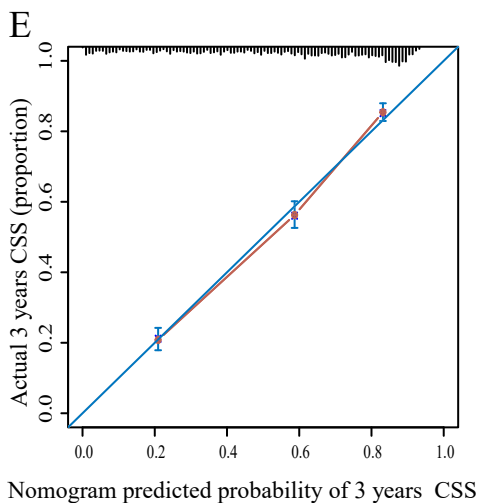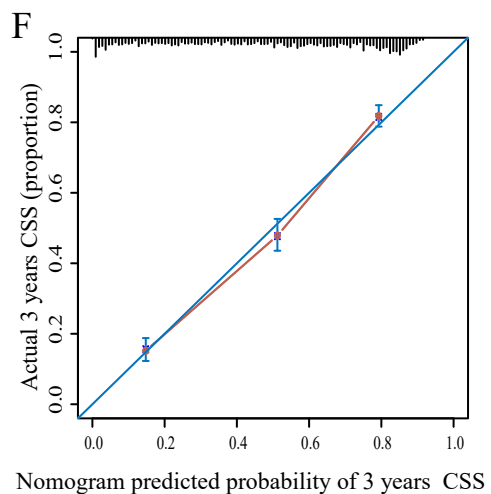

Supplement: Supplementary file 2 — Supplementary Figure S2: Performance of the prognostic nomogram model in the missing data cohort for CSS (PDF 430 KB) [file 432_2021_3654_MOESM2_ESM.pdf]
