# Supplementary material for: The log odds of negative lymph nodes/T stage: a new prognostic and predictive tool for resected gastric cancer patients
Source: J Cancer Res Clin Oncol. 2021 May 18;147(8):2259–69. doi: 10.1007/s00432-021-03654-y (PMC8236481; doi:10.1007/s00432-021-03654-y)
Supplement: Supplementary file 3 — Supplementary Table S1: Baseline clinicopathological features of patients in the excluded cohort and included cohort; Table S2: Univariate analysis of included variables on overall survival; Table S3: Multivariate analysis of prognostic factors for overall survival; Table S4: Multivariate analyses for evaluating LONT effect on overall survival based on different clinicopathological factors; Table S5: Discriminatory ability of prognostic factors in predicting overall survival in the validation cohort. (DOCX 34 KB) [file 432_2021_3654_MOESM3_ESM.docx]

Table 1. Baseline clinicopathological features of patients in the excluded cohort and included cohort

| **Feature** | **Excluded (%)** | **Included (%)** | ***p-value*** |
| --- | --- | --- | --- |
|  | **N=15160** | **N=2799** |  |
| Median LONT (IQR) | 0.7 (0.4-1.0) | 0.85 (0.48-1.11) | <0.001 |
| Median PLN count (IQR) | 1 (0-5) | 0 (0-4) | <0.001 |
| Median ELN count (IQR) | 16 (9-24) | 15(8-22) | 0.001 |
| Median NLN count (IQR) | 12 (6-20) | 12 (5-19) | <0.001 |
| Age (years) |  |  | <0.001 |
| ＜60 | 4235(28.5) | 1111(40) |  |
| ≥60 | 10835(71.25) | 1668(60) |  |
| **Sex** |  |  | 0.219 |
| Male | 9673(63.8) | 1807(65.0) |  |
| Female | 5487(36.2) | 972(35.0) |  |
| **TNM stage** |  |  | <0.001 |
| I | 4482(29.6) | 1001(55.6) |  |
| II | 4813(31.7) | 667(31.2) |  |
| III | 5856(38.7) | 911(32.8) |  |
| **T stage** |  |  | <0.001 |
| T1 | 3508(23.1) | 845(30.4) |  |
| T2 | 2166(14.3) | 296(10.7) |  |
| T3 | 59279(39.1) | 1002(36.1) |  |
| T4a | 2791(18.4) | 479(17.2) |  |
| T4b | 768(5.1) | 157(5.6) |  |
| **N stage** |  |  | <0.001 |
| N0 | 6761(44.6) | 1546(55.6) |  |
| N1 | 2807(18.5) | 403(14.5) |  |
| N2 | 2572(17.0) | 369(11.3) |  |
| N3a | 2156(14.2) | 306(11.0) |  |
| N3b | 864(5.7) | 155(5.6) |  |
| **Location** |  |  | <0.001 |
| Cardiac/fundus | 4067(26.8) | 1019(36.7) |  |
| Body | 4382(28.9) | 593(22.3) |  |
| Antrum/pylorus | 4782(31.5) | 629(22.6) |  |
| other | 1929(12.7) | 538(19.4) |  |
| **Histology type** |  |  | <0.001 |
| Adenocarcinoma | 11971(79.0) | 1894(68.2) |  |
| MAC and SRCC | 3189(21.0) | 885(31.8) |  |
| **Race** |  |  | <0.001 |
| white | 9840(64.9) | 1917(69.0) |  |
| Black | 1969(13.0) | 345(12.4) |  |
| Others | 3351 (22.1) | 447(16.1) |  |
| Unknow | Na | 70（2.5） |  |

Abbreviation: ELNs, examined lymph nodes; IQR, interquartile range; LONT, Log odds of negative lymph nodes/T-stage; MAC, mucinous adenocarcinoma; NLNs, negative lymph nodes; SRCC, signet-ring cell carcinoma.

Table S2: Univariate analysis of included variables on overall survival

| **Variable** | **Training cohort (*n* = 10612)** | | | **Validation cohort (*n* = 4548)** | | |
| --- | --- | --- | --- | --- | --- | --- |
|  | **HR** | **95%CI** | ***P*** | **HR** | **95%CI** | ***P*** |
| Tumor size^1^ | 1.003 | 1.003-1.004 | <0.001 | 1.003 | 1.002-1.003 | <0.001 |
| LONS^1^ | 0.366 | 0.348-0.385 | <0.001 | 0.381 | 0.353-0.411 | <0.001 |
| LONT^1^ | 0.308 | 0.282 - 0.325 | <0.001 | 0.333 | 0.307-0.360 | <0.001 |
| Median PLN count (IQR) | 1.057 | 1.054-1.060 | <0.001 | 1.051 | 1.046-1.055 | <0.001 |
| Median ELN count (IQR) | 0.994 | 0.992-0.996 | <0.001 | 0.991 | 0.988-0.995 | <0.001 |
| Median NLN count (IQR) | 0.968 | 0.965-0.971 | <0.001 | 0.968 | 0.964-0.972 | <0.001 |
| Age(ref = ≥60) | 0.704 | 0.664-0.747 | <0.001 | 0.692 | 0.631-0.758 | <0.001 |
| Sex (ref = male) | 0.931 | 0.883-0.982 | 0.009 | 0.979 | 0.904-1.061 | 0.611 |
| **TNM (ref = stage III)** |  |  |  |  |  |  |
| I | 0.261 | 0.244-0.280 | <0.001 | 0.296 | 0.267-0.329 | <0.001 |
| II | 0.486 | 0.458 -0.515 | <0.001 | 0.550 | 0.502-0.601 | <0.001 |
| **T stage (ref = T4b)** |  |  | <0.001 |  |  | <0.001 |
| T1 | 0.228 | 0.203-0.257 | <0.001 | 0.236 | 0.196-0.285 | <0.001 |
| T2 | 0.358 | 0.318-0.403 | <0.001 | 0.367 | 0.303-0.444 | <0.001 |
| **T3** | 0.590 | 0.533-0.653 | <0.001 | 0.598 | 0.505-0.707 | <0.001 |
| T4a | 0.857 | 0.770-0.953 | 0.004 | 0.837 | 0.702-0.998 | 0.048 |
| **N stage ( ref = N3b)** |  |  | <0.001 |  |  | <0.001 |
| N0 | 0.215 | 0.194-0.237 | <0.001 | 0.239 | 0.205-0.278 | <0.001 |
| N1 | 0.371 | 0.334-0.412 | <0.001 | 0.428 | 0.364-0.503 | <0.001 |
| N2 | 0.502 | 0.452-0.557 | <0.001 | 0.513 | 0.437-0.603 | <0.001 |
| N3a | 0.675 | 0.608-0.749 | <0.001 | 0.774 | 0.659-0.910 | 0.002 |
| **Differentiation (ref =G3+G4)** |  |  |  |  |  |  |
| G1 | 0.526 | 0.461-0.602 | <0.001 | 0.630 | 0.520-0.762 | <0.001 |
| G2 | 0.735 | 0.694-0.778 | <0.001 | 0.779 | 0.715-0.850 | <0.001 |
| Histology (ref=adenocarcinoma) | 1.120 | 1.055-1.189 | <0.001 | 1.086 | 0.992-1.190 | 0.074 |
| Location (ref =Cardiac) |  |  |  |  |  |  |
| Middle^2^ | 0.833 | 0.777-0.892 | <0.001 | 0.846 | 0.731-0.9279 | 0.025 |
| Antrum/pylorus | 0.932 | 0.872-0.996 | 0.037 | 0.855 | 0.775-0.944 | 0.002 |
| other | 1.165 | 1.073-1.266 | <0.001 | 1.007 | 0.887-1.144 | 0.912 |
| **Race (ref =others)** |  |  |  |  |  |  |
| white | 1.332 | 1.247-1.422 | <0.001 | 1.406 | 1.268-1.559 | <0.001 |
| Black | 1.356 | 1.239-1.483 | <0.001 | 1.500 | 1.306-1.722 | <0.001 |

^1^These variables were treated as continuous data; ^2^The location included the body, fundus, greater curvature and lesser curvature of the stomach.

Abbreviation: CI: Confidence interval; HR, Hazard ratio; LONT, Log odds of negative lymph nodes/T-stage, NLNs, negative lymph nodes; ref: Reference.

Table S3: Multivariate analysis of prognostic factors for overall survival

| **Variable** | **Training cohort (*n* = 10612)** | | | **Validation cohort (*n* = 4548)** | | |
| --- | --- | --- | --- | --- | --- | --- |
|  | **HR** | **95%CI** | ***P*** | **HR** | **95%CI** | ***P*** |
| Age(ref = >=60 years old) | 0.600 | 0.565-0.638 | <0.001 | 0.615 | 0.560-0.676 | <0.001 |
| Tumor size^1^ | 1.001 | 1.001-1.002 | <0.001 | 1.001 | 1.00-1.002 | 0.045 |
| LONT^1^ | 0.480 | 0.450-0.513 | <0.001 | 0.488 | 0.442-0.538 | <0.001 |
| Sex (ref = male) | 0.965 | 0.914-1.019 | 0.200 | 1.000 | 0.920-1.087 | 0.997 |
| **T stage (ref = T4b)** |  |  |  |  |  |  |
| T1 | 0.594 | 0.519-0.678 | <0.001 | 0.601 | 0.484-0.748 | <0.001 |
| T2 | 0.648 | 0.571-0.735 | <0.001 | 0.665 | 0.542-0.817 | <0.001 |
| T3 | 0.777 | 0.700-0.863 | <0.001 | 0.760 | 0.638-0.905 | 0.002 |
| T4a | 0.877 | 0.788-0.977 | 0.017 | 0.865 | 0.723-1.034 | 0.122 |
| **N stage ( ref = N3b)** |  |  |  |  |  |  |
| N0 | 0.359 | 0.322-0.401 | <0.001 | 0.374 | 0.316-0.442 | <0.001 |
| N1 | 0.502 | 0.449-0.561 | <0.001 | 0.522 | 0.442-0.618 | <0.001 |
| N2 | 0.612 | 0.550-0.682 | <0.001 | 0.587 | 0.498-0.692 | <0.001 |
| N3a | 0.768 | 0.691-0.854 | <0.001 | 0.843 | 0.715-0.992 | 0.040 |
| **Differentiation (ref =G3+G4)** |  |  |  |  |  | 0.262 |
| G1 | 0.844 | 0.735-0.970 | 0.017 | 0.963 | 0.788-1.176 | 0.711 |
| G2 | 0.891 | 0.838-0.947 | <0.001 | 0..926 | 0.845-1.015 | 0.102 |
| Histology(ref =MAC and SRCC) | 0.998 | 0.936-1.065 | 0.951 | 0.982 | 0.891-1.081 | 0.707 |
| Location (ref =Cardiac) |  |  |  |  |  |  |
| Middle^2^ | 0.753 | 0.701-0.810 | <0.001 | 0.822 | 0.737-0.917 | <0.001 |
| Antrum/pylorus | 0.809 | 0.754-0.869 | <0.001 | 0.767 | 0.689-0.855 | <0.001 |
| other | 0.858 | 0.787-0.936 | 0.001 | 0.776 | 0.678-0.888 | 0.003 |
| **Race (ref =others)** |  |  |  |  |  |  |
| white | 1.266 | 1.183-1.354 | <0.001 | 1.261 | 1.134-1.402 | <0.001 |
| Black | 1.338 | 1.223-1.465 | <0.001 | 1.393 | 1.210-1.602 | <0.001 |

^1^These variables were treated as continuous data; ^2^The location included the body, fundus, greater curvature and lesser curvature of the stomach.

Abbreviation: CI: Confidence interval; HR, Hazard ratio; LONT, Log odds of negative lymph nodes/T-stage; ref: Reference.

Table S4: Multivariate analyses for evaluating LONT effect on overall survival based on different clinicopathological factors^1^

| **Variable** | **Training cohort (*n* = 10612)** | | | **Validation cohort (*n* = 4548)** | | |
| --- | --- | --- | --- | --- | --- | --- |
|  | **HR** | **95%CI** | ***P^b^*** | **HR** | **95%CI** | ***P^b^*** |
| All | 0.432 | 0.402-0.463 | <0.001 | 0.458 | 0.417-0.503 | <0.001 |
| **N stage** |  |  |  |  |  |  |
| N0 | 0.443 | 0.389-0.504 | <0.001 | 0.478 | 0.392-0.582 | <0.001 |
| N1 | 0.423 | 0.366-0.489 | <0.001 | 0.484 | 0.392-0.599 | <0.001 |
| N2 | 0.452 | 0.393-0.520 | <0.001 | 0.491 | 0.400-0.603 | <0.001 |
| N3a | 0.488 | 0.428-0.555 | <0.001 | 0.436 | 0.356-0.532 | <0.001 |
| N3b | 0.543 | 0.452-0.653 | <0.001 | 0.357 | 0.262-0.488 | <0.001 |
| **T stage** |  |  |  |  |  |  |
| T1 | 0.479 | 0.391-0.587 | <0.001 | 0.435 | 0.318-0.595 | <0.001 |
| T2 | 0.466 | 0.374-0.582 | <0.001 | 0.513 | 0.375-0.702 | <0.001 |
| T3 | 0.450 | 0.407-0.498 | <0.001 | 0.474 | 0.407-0.552 | <0.001 |
| T4a | 0.491 | 0.434-0.555 | <0.001 | 0.457 | 0.378-0.551 | <0.001 |
| T4b | 0.371 | 0.297-0.463 | <0.001 | 0.465 | 0.323-0.668 | <0.001 |
| **TNM stage** |  |  |  |  |  |  |
| I | 0.503 | 0.425-0.595 | <0.001 | 0.492 | 0.384=0.631 | <0.001 |
| II | 0.453 | 0.399-0.513 | <0.001 | 0.486 | 0.401-0.589 | <0.001 |
| III | 0.456 | 0.421-0.494 | <0.001 | 0.461 | 0.409-0.521 | <0.001 |
| **Sex** |  |  |  |  |  |  |
| Male | 0.486 | 0.449-0.526 | <0.001 | 0.453 | 0.403-0.509 | <0.001 |
| Female | 0.403 | 0.363-0.447 | <0.001 | 0.463 | 0.358-0.533 | <0.001 |
| **Age years** |  |  |  |  |  |  |
| ≤60 | 0.405 | 0.358-0.458 | <0.001 | 0.417 | 0.344-0.505 | <0.001 |
| >60 | 0.476 | 0.442-0.512 | <0.001 | 0.460 | 0.413-0.513 | <0.001 |
| **Differentiation** |  |  |  |  |  |  |
| G1 | 0.659 | 0.452-0.961 | 0.030 | 0.468 | 0.274-0.798 | 0.005 |
| G2 | 0.422 | 0.371-0.480 | <0.001 | 0.485 | 0.404-0.582 | <0.001 |
| G3 | 0460 | 0.428-0.495 | <0.001 | 0.452 | 0.404-0.506 | <0.001 |
| **Location** |  |  |  |  |  |  |
| Cardiac | 0.524 | 0.463-0.592 | <0.001 | 0.471 | 0.392-0.565 | <0.001 |
| Non-Cardiac | 0.425 | 0.391-0.462 | <0.001 | 0.443 | 0.392-0.501 | <0.001 |
| **Race** |  |  |  |  |  |  |
| white | 0.461 | 0.427-0.499 | <0.001 | 0.453 | 0.404-0.509 | <0.001 |
| Black | 0.459 | 0.388-0.543 | <0.001 | 0.516 | 0.404-0.659 | <0.001 |
| Others | 0.443 | 0.385-0.510 | <0.001 | 0.423 | 0.339-0.529 | <0.001 |
| Histology |  |  |  |  |  |  |
| Adenocarcinoma | 0.461 | 0.428-0.497 | <0.001 | 0.457 | 0.409-0.510 | <0.001 |
| MAC and SRCC) | 0.451 | 0.513-0.667 | <0.001 | 0.474 | 0.396-0.567 | <0.001 |
| ELNs |  |  |  |  |  |  |
| 10 | 0.478 | 0.410-0.558 | <0.001 | 0.561 | 0.444-0.708 | <0.001 |
| 11-20 | 0.347 | 0.297-0.403 | <0.001 | 0.269 | 0.210-0.345 | <0.001 |
| >20 | 0.325 | 0.282-0.374 | <0.001 | 0.305 | 0.243-0.383 | <0.001 |

^1^LONT was treated as continuous data，and adjusted by age, race, sex, location, histology type, differention and TNM stage.

Abbreviation: CI: Confidence interval; HR, Hazard ratio; LONT, Log odds of negative lymph nodes/T-stage; MAC, mucinous adenocarcinoma; SRCC, signet-ring cell carcinoma.

Table S5: Discriminatory ability of prognostic factors in predicting overall survival in the validation cohort.

| **Variable** | **Validation cohort (*n* = 4548)** | |
| --- | --- | --- |
|  | **C-index** | ***AUC*** |
| Age | 0.536 | 0.534 |
| Tumor size | 0.605 | 0.642 |
| LONT | 0.668 | 0.719 |
| TNM | 0.685 | 0.723 |
| T stage | 0.642 | 0.701 |
| N stage | 0.650 | 0.703 |
| Differentiation | 0.552 | 0.572 |
| Location | 0.533 | 0.550 |
| Race | 0.528 | 0.539 |
| LONT+Tstage+Nstage+Location+Age  +Grade+size+race | 0.714 | 0.779 |
| LONT+Tstage+Nstage+Location+Age  +race+size | 0.713 | 0.777 |
| LONT+Tstage+Nstage+Location+Age  +race | 0.712 | 0.777 |
| LONT+Tstage+Nstage+Location+Age | 0.710 | 0.776 |
| LONT+Tstage+Nstage+Location | 0.702 | 0.770 |
| LONT+Tstage+Nstage | 0.699 | 0.765 |
| LONT+Nstage | 0.696 | 0.758 |
| LONT+Tstage | 0.68 | 0.741 |

LONT was treated as continuous data.

Abbreviation: AUC, the area under the time-dependent receiver operating characteristic curve; C-index, concordance index; LONT, Log odds of negative lymph nodes/T-stage.
